# Supplementary figures and images for: State-Dependent Motor Cortex Stimulation Reveals Distinct Mechanisms for Corticospinal Excitability and Cortical Responses
Source: eNeuro. 2024 Nov 26;11(11):ENEURO.0450-24.2024. doi: 10.1523/ENEURO.0450-24.2024 (PMC11595597; doi:10.1523/ENEURO.0450-24.2024)

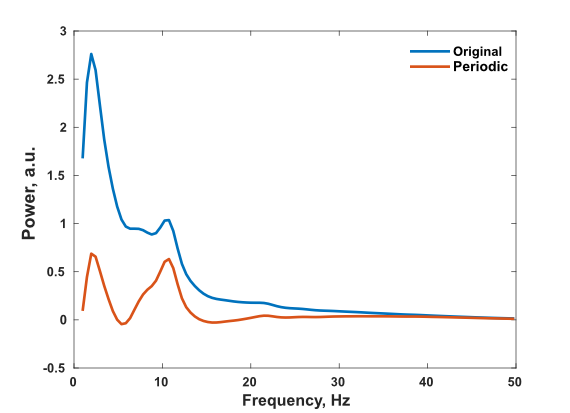

Supplement: Figure 1-1 — Global pre-TMS power spectrum. The original power spectrum and the periodic power spectrum calculated by irregular resampling auto-spectral analysis (IRASA) in the 500 ms window prior to TMS delivery. There is dominant mu activity and low beta activity. Download Figure 1-1, TIF file. [file eneuro-11-ENEURO.0450-24.2024-s002.tif]

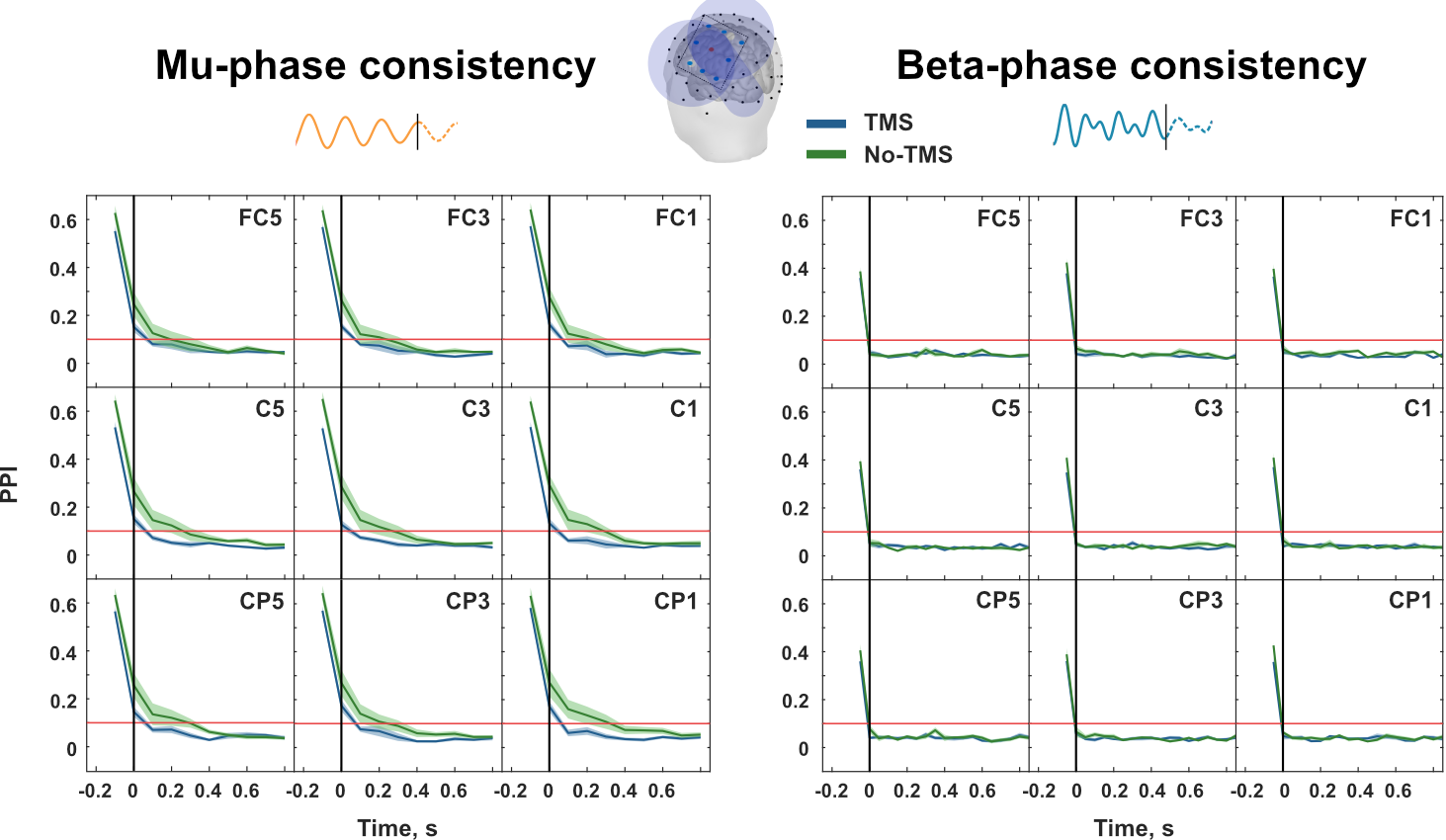

Supplement: Figure 1-2 — Phase Preservation Index (PPI) for phase specific targeting. PPI values are calculated with reference to mu phase at 100 ms prior to TMS pulse, at 100 ms intervals. Blue trace indicates the PPI values for real TMS, and green trace indicates PPI for TMS trigger without actual pulse. Shaded region depicts the standard error of mean (SEM) of PPI. Plots for all electrodes in the Laplacian montage (FC5, FC3, FC1, C5, C3, C1, CP5, CP3 and CP1) are shown here. Black vertical line indicates the TMS trigger, and the red horizontal line (PPI = 0.1) indicates the threshold for phase preservation calculated according to Fischer, 1993. Phase preservation for mu (left) and beta (right) are shown here. For real TMS condition, there is no substantial evidence for mu or beta phase preservation after TMS delivery. Download Figure 1-2, TIF file. [file eneuro-11-ENEURO.0450-24.2024-s003.tif]

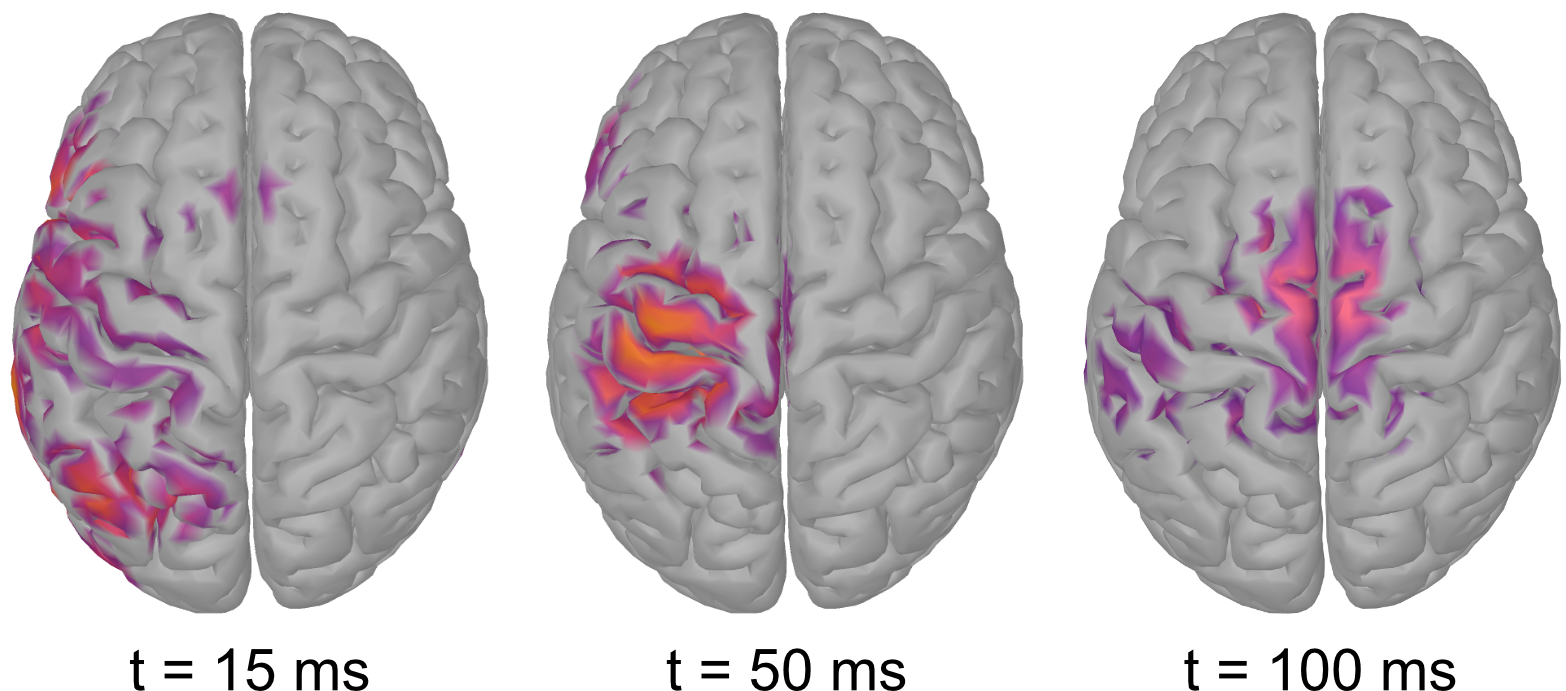

Supplement: Figure 4-1 — EEG sources computed at latencies of TEP components. EEG source reconstruction by minimum norm estimate show distributed activity in motor, somatosensory association and auditory cortices the ipsilateral hemisphere at = 15 ms. At t = 50 ms, the activity is localized to the ipsilateral motor cortex at t = 50 ms. At t = 100 ms the source activity is distributed bilaterally in the motor and somatosensory areas. The spatial distribution of the sources that generate the TEP responses could partially explain the discrepancy between TEP-MEP phase relationship. Download Figure 4-1, TIF file. [file eneuro-11-ENEURO.0450-24.2024-s004.tif]
